# Prevalence and Clinical Correlates of Psychiatric Disorders in a Hispanic-Majority Inflammatory Bowel Disease Cohort

**DOI:** 10.7759/cureus.108438

**Published:** 2026-05-07

**Authors:** Matthew Orosa, Mindy Xu, Andrew Chang

**Affiliations:** 1 Internal Medicine, Riverside University Health System Medical Center, Moreno Valley, USA; 2 Psychiatry, Riverside University Health System Medical Center, Moreno Valley , USA; 3 Gastroenterology and Hepatology, Loma Linda University School of Medicine, Loma Linda, USA

**Keywords:** anxiety, depression, hospitalization, inflammatory bowel disease, psychiatric comorbidity

## Abstract

Introduction: Inflammatory bowel disease (IBD) is a chronic immune-mediated condition associated with a substantial burden of psychiatric comorbidity. Depression, anxiety, and other psychiatric disorders are increasingly recognized as important contributors to disease activity, healthcare utilization, and quality of life. However, existing data are largely derived from tertiary care populations, with limited representation of underserved and Hispanic patients.

Methods: We conducted a single-center, retrospective cohort study of adult patients with IBD within a county health system from 2015 to 2024. The primary outcome was the presence of at least one psychiatric disorder, defined as depression, anxiety, post-traumatic stress disorder, bipolar disorder, psychotic disorders, substance use disorders, or insomnia. Demographic and clinical variables, including age, sex, IBD type, smoking status, and IBD-related hospitalization, were analyzed. Associations were evaluated using Mann-Whitney U testing, chi-square testing, and multivariable logistic regression.

Results: A total of 158 patients met the inclusion criteria. The prevalence of at least one psychiatric disorder was 48.7%. Depression and anxiety were the most common diagnoses (30.4% each), followed by insomnia (26.6%). The IBD-related hospitalization was significantly associated with psychiatric comorbidity in univariate analysis (54.5% vs. 30.9%, p=0.004). In multivariable analysis, hospitalization was associated with numerically increased odds that did not reach conventional statistical significance (OR 2.32, 95% CI 0.98-5.50, p=0.056).

Conclusions: Psychiatric disorders are highly prevalent among IBD patients in a county health system, affecting nearly half of the cohort. The IBD-related hospitalization was associated with psychiatric comorbidity in univariate analysis and showed a non-significant trend in multivariable analysis. The results point to the value of coordinated, team-based care models tailored to the needs of underserved communities.

## Introduction

Inflammatory bowel disease (IBD), encompassing Crohn’s disease and ulcerative colitis, is a chronic relapsing condition characterized by gastrointestinal inflammation and systemic manifestations. In addition to its physical burden, IBD is increasingly recognized as a disorder with significant psychological and psychiatric implications. Depression and anxiety are among the most frequently reported psychiatric comorbidities and have been consistently associated with impaired quality of life, reduced treatment adherence, and worse clinical outcomes [1].

The pathophysiological relationship between IBD and psychiatric disorders is complex and bidirectional. The brain-gut axis, a network involving neural, hormonal, and immune pathways, plays a central role in mediating this interaction [2]. Psychological stress and mood disorders may influence intestinal inflammation through immune modulation and alterations in gut permeability, while chronic inflammation and symptom burden may contribute to the development or exacerbation of psychiatric illness.

Emerging evidence suggests that psychiatric comorbidities are not merely a consequence of chronic disease but may actively contribute to disease progression. Prospective studies have demonstrated that anxiety and depression are associated with increased risk of disease flares and symptom exacerbation [3]. Furthermore, psychiatric disorders have been linked to increased healthcare utilization, including higher rates of emergency department visits, hospitalizations, and corticosteroid use [4,5].

Despite the growing recognition of psychiatric comorbidities in IBD, most studies have been conducted in tertiary care settings with relatively homogeneous patient populations. County health systems, which serve a disproportionate number of underserved patients, often care for individuals with higher psychosocial stress, increased prevalence of substance use, and limited access to mental health services [6]. Hispanic populations, in particular, remain underrepresented in IBD research. A 2025 study from the National Inpatient Sample highlights that despite being the largest minority group in the United States, Hispanics remain significantly underrepresented in IBD research [7]. 

Given these gaps, there is a need to better characterize the burden and correlates of psychiatric disorders in real-world, underserved populations. The primary objective of this retrospective study is to evaluate the prevalence and clinical associations of psychiatric comorbidities in a predominantly Hispanic IBD population within a county health system.

## Materials and methods

Study design and population

This retrospective cohort study was conducted within a county health system and included adult patients aged 18 years and older with a diagnosis of inflammatory bowel disease (IBD) between January 1, 2015, and December 31, 2024. Patients were identified using the International Classification of Diseases, tenth revision (ICD-10) codes [8] (K50.x, K51.x) for Crohn’s disease and ulcerative colitis. Eligible patients were required to have at least one documented healthcare encounter within the study period to ensure adequate clinical data capture.

Inclusion and exclusion criteria

Patients were included if they met the following criteria: age ≥18 years, a documented diagnosis of IBD based on ICD-10 coding, and sufficient electronic medical record (EMR) data to ascertain psychiatric status and relevant clinical variables. Sufficient EMR data were defined as the presence of at least one clinical encounter within the study period with complete documentation of demographic variables and diagnostic coding sufficient to determine both IBD status and the presence or absence of psychiatric disorders. Patients lacking documentation of psychiatric diagnoses or key covariates were excluded. Patients were excluded if they had incomplete or missing data, lacked sufficient documentation to determine the presence or absence of psychiatric disorders, or had an unclear or inconsistent diagnosis of IBD. 

Outcome definition

The primary outcome of interest was the presence of at least one psychiatric disorder. Secondary objectives included identifying clinical factors associated with psychiatric comorbidity. Psychiatric diagnoses were identified using ICD-10 codes, including depression (F32.x, F33.x), anxiety disorders (F40.x-F41.x), post-traumatic stress disorder (F43.1), bipolar disorder (F31.x), psychotic disorders (F20.x, F22.x-F23.x, F25.x, F28-F29), substance use disorders (F10.x-F19.x, excluding tobacco use disorder), and insomnia (F51.0, G47.0x). The primary outcome (presence of at least one psychiatric disorder) was defined as a binary variable; however, individual psychiatric conditions were not mutually exclusive, and patients could be counted in multiple diagnostic categories. Insomnia was included as a psychiatric comorbidity due to its high prevalence in patients with IBD and its clinical association with psychological distress, reduced quality of life, and disease activity. Although insomnia may be multifactorial and can occur secondary to pain, corticosteroid use, or nocturnal gastrointestinal symptoms, it was included to capture the broader burden of neuropsychiatric and sleep-related disturbances in this population.

Predictor variables

Predictor variables included demographic and clinical characteristics hypothesized to be associated with psychiatric comorbidity. These included age (modeled as a continuous variable), sex, IBD type (Crohn’s disease versus ulcerative colitis), tobacco use (categorized as current versus former versus never smoker), and IBD-related hospitalization. The IBD-related hospitalization was defined as at least one inpatient admission during the study period with a primary discharge diagnosis of IBD (ICD-10 K50.x or K51.x), as identified through the EMR. It served as a surrogate marker for disease severity and healthcare utilization rather than a direct measure of disease severity. Admissions were considered IBD-related if associated with disease activity or management, including flares, complications, surgical interventions, infections, or medication-related adverse effects. The temporal relationship between hospitalization and psychiatric diagnosis (i.e., whether hospitalization occurred before or after the psychiatric diagnosis) was not assessed, and hospitalization was therefore treated as a non-time-dependent variable.

Data collection

Data were extracted from the EMR and de-identified prior to analysis. The study was approved by the Institutional Review Board (IRB) of Riverside University Health System (approval no. 2412576-2). Extracted variables included demographic characteristics, clinical diagnoses, and hospitalization history. Data quality checks were performed to ensure completeness and consistency, and duplicate entries were removed where identified. All data were stored securely on institutional servers in accordance with applicable privacy and confidentiality standards.

Statistical analysis

Descriptive statistics were used to summarize baseline demographic and clinical characteristics. Continuous data were summarized using medians and analyzed with the Mann-Whitney U test given their non-normal distribution. Categorical data were expressed as frequencies and percentages, with group comparisons performed using either the chi-square test or Fisher’s exact test, as appropriate. Univariate analyses were performed to assess associations between individual predictor variables and the presence of psychiatric disorders. 

Variables included in the multivariable logistic regression model were selected based on clinical relevance and prior literature demonstrating associations with psychiatric comorbidity in patients with inflammatory bowel disease. These included age, sex, IBD type (Crohn’s disease vs. ulcerative colitis), tobacco use, and IBD-related hospitalization. The sample size was considered adequate for multivariable logistic regression based on the commonly accepted rule of at least 10 outcome events per predictor variable. In this study, 77 patients had psychiatric disorders, allowing for the inclusion of up to seven predictor variables without substantial risk of model overfitting. As the final model included five predictor variables, the analysis was adequately powered. Odds ratios (ORs) with 95% CI were reported. A two-tailed p-value <0.05 was considered statistically significant.

## Results

Patient characteristics

A total of 158 patients met the inclusion criteria. The cohort was of Hispanic-majority. Psychiatric disorders were present in 77 patients (48.7%), indicating that nearly half of the patients carried at least one psychiatric diagnosis. Baseline demographic and clinical characteristics of the study cohort are displayed in Table 1. 

**Table 1:** Baseline demographic and clinical characteristics of the study cohort Data are presented as median (interquartile range) or counts and percentages. IBD: Inflammatory bowel disease

Variable	Overall (n = 158)
Age, median (IQR)	45 (35.0-56.0)
Female, n (%)	69 (43.7)
Male, n (%)	89 (56.3)
Hispanic ethnicity, n (%)	83 (52.5)
Non-Hispanic ethnicity, n (%)	75 (47.5)
Crohn’s disease, n (%)	45 (28.5)
Ulcerative colitis, n (%)	113 (71.5)
Tobacco use (ever), n (%)	48 (30.4)
IBD-related hospitalization	67 (42.4)

Prevalence of psychiatric disorders

Depression and anxiety were the most common psychiatric conditions, each affecting 48 patients (30.4%). Insomnia was present in 42 patients (26.6%), followed by substance use disorder in 13 patients (8.2%), bipolar disorder in 10 patients (6.3%), post-traumatic stress disorder in eight patients (5.1%), and psychotic disorders in five patients (3.2%). Results are displayed in Table 2. 

**Table 2:** Prevalence of psychiatric disorders

Psychiatric disorder	Prevalence, n (%)
Depression	48 (30.4)
Anxiety	48 (30.4)
Insomnia	42 (26.6)
Substance use disorder	13 (8.2)
Bipolar disorder	10 (6.3)
Post-traumatic stress disorder	8 (5.1)
Psychotic disorders	5 (3.2)

Univariate analysis

Inflammatory bowel disease-related hospitalization was more common among patients with psychiatric disorders compared to those without (42 patients (54.5%) vs. 25 patients (30.9%), p=0.004). There were no statistically significant differences in psychiatric disorder prevalence based on age, sex, IBD type, or smoking status (Table 3). 

**Table 3:** Univariate analysis of factors associated with psychiatric disorders Continuous variables were compared using the Mann-Whitney U test, and categorical variables were compared using the chi-square or Fisher’s exact tests. IBD: Inflammatory bowel disease

Variable	Psychiatric disorder present (n =77)	Psychiatric disorder not present (n=81)	p-value
Age (median)	45.0	46.0	0.8008
Female, n (%)	37 (48.1)	32 (39.5)	0.3187
Male, n (%)	40 (51.9)	49 (60.5)	0.3187
Crohn’s disease, n (%)	23 (29.9)	22 (27.2)	0.9323
Tobacco use (ever), n (%)	24 (31.5)	24 (29.6)	0.9476
IBD hospitalization, n (%)	42 (54.5)	25 (30.9)	0.0044

Multivariable analysis

In multivariable logistic regression, IBD-related hospitalization showed a strong trend toward association with psychiatric comorbidity, with more than a two-fold increase in odds (OR 2.32, 95% CI 0.98-5.50), although this did not reach conventional statistical significance (p=0.056). No other variables demonstrated significant associations (Table 4). 

**Table 4:** Multivariable analysis of factors associated with psychiatric disorders Multivariable logistic regression analysis was performed to evaluate factors associated with psychiatric disorders. Odds ratios (ORs) with 95% CI are reported. The p-values were derived from the logistic regression model. A two-tailed p-value <0.05 was considered statistically significant. Reference categories are indicated. IBD: Inflammatory bowel disease

Variable	OR	95% CI	p-value
Age (per year increase)	1.01	0.98-1.04	0.6431
Female	1.16	0.49-2.75	0.7411
Male	1.00	Reference	Reference
Crohn’s	1.33	0.55-3.22	0.5218
Tobacco use	1.53	0.64-3.71	0.3413
IBD hospitalization	2.32	0.98-5.50	0.0557

## Discussion

In this retrospective cohort, nearly half of the patients (48.7%) were found to have at least one psychiatric disorder, with anxiety and depression each present in approximately 30% of the cohort. This high prevalence is also consistent with prior literature demonstrating a substantial burden of psychiatric comorbidity in IBD populations [1,5,9]. Prior work has highlighted the benefits of multidisciplinary approaches, including improved symptom control, reduced healthcare utilization, and enhanced patient satisfaction [10-12]. Furthermore, the rates of psychiatric disorders in this study are notably higher than those reported in the general U.S. adult population. Recent National Health Interview Survey data estimate that approximately 21.4% of adults report symptoms of depression and 18.2% report symptoms of anxiety over two weeks using validated screening instruments [13]. The predominance of depression and anxiety observed in this study further aligns with existing evidence and underscores their central role in the psychosocial burden of IBD.

One of the most notable findings of this study is the strong association between IBD-related hospitalization and psychiatric comorbidity. Patients with a history of hospitalization had significantly higher rates of psychiatric disorders in univariate analysis, and this association remained robust in adjusted analyses, with a more than two-fold increase in odds. Although the multivariable analysis did not reach statistical significance, possibly due to limited sample size, the magnitude and consistency of the association suggest a clinically meaningful relationship.

These findings are consistent with prior studies demonstrating that psychiatric disorders are associated with increased healthcare utilization and more severe disease phenotypes in IBD [4]. The relationship between hospitalization and psychiatric comorbidity may reflect both the impact of severe disease on mental health and the potential for psychiatric conditions to worsen disease outcomes through behavioral and physiological mechanisms.

The bidirectional nature of the brain-gut axis provides a plausible explanation for these findings. Psychological stress and mood disorders may exacerbate intestinal inflammation through immune-mediated pathways, while recurrent disease flares and hospitalizations may contribute to the development or worsening of psychiatric conditions [3]. This interplay highlights the importance of addressing both physical and mental health in the management of IBD.

Importantly, this study contributes to the limited body of literature focusing on underserved and predominantly Hispanic populations. Individuals in these groups frequently encounter obstacles to obtaining mental health services, such as financial constraints, language differences, and restricted access to healthcare resources [14,15]. The high prevalence of psychiatric disorders observed in this study likely reflects a true increased burden in this population. However, because psychiatric diagnoses were identified using the ICD-10 codes [8], under-recognition or under-documentation of psychiatric conditions may have led to an underestimation of the true prevalence. 

The findings of this study support the growing emphasis on integrated care models that incorporate behavioral health into the management of IBD. The implementation of routine screening for depression and anxiety, referral pathways, behavioral health integration, social work involvement, and culturally/language-concordant mental health resources may be particularly impactful. 

Limitations

This study has several limitations. Its retrospective design limits the ability to establish causal relationships. The study did not include objective IBD activity measures, medication exposure, corticosteroid use, biologic therapy, endoscopic severity, inflammatory markers, surgery history, insurance status, language preference, or socioeconomic factors, all of which may confound the relationship between psychiatric comorbidity and hospitalization. Reliance on ICD-10 coding [8] may result in underestimation of psychiatric diagnoses, particularly for conditions that are underdiagnosed or underreported. There is also the likelihood that patients with more healthcare encounters may be more likely to receive psychiatric diagnoses. The sample size may also limit statistical power, particularly in multivariable analyses. Although the number of outcome events (n=77) allowed for inclusion of multiple predictors, the model remains susceptible to overfitting, and the estimates may be unstable. In particular, the association between IBD-related hospitalization and psychiatric disorders approached but did not meet conventional statistical significance (p=0.056), suggesting that this finding should be interpreted with caution. Finally, while the focus on a Hispanic-majority population is a strength, it may limit generalizability to other settings.

## Conclusions

Psychiatric disorders are highly prevalent among patients with IBD in the county health system where this retrospective study was conducted, affecting nearly half of the cohort. The IBD-related hospitalizations were associated with psychiatric comorbidity in univariate analysis and demonstrated a non-significant trend after adjustment. These results underscore the need for consistent mental health screening and closer incorporation of psychiatric care into IBD management, especially for underserved populations.
